# Superantigens and SARS-CoV-2

**DOI:** 10.3390/pathogens11040390

**Published:** 2022-03-23

**Authors:** Adam Hamdy, Anthony Leonardi

**Affiliations:** 1 Panres Pandemic Research, Newport TF10 8PG, UK; 2 Johns Hopkins Bloomberg School of Public Health, Johns Hopkins University, Baltimore, MD 21205, USA; aleona10@jh.edu

**Keywords:** SARS-CoV-2, COVID-19, long COVID, post-acute COVID-19 syndrome, reinfection, coronavirus, neuroinvasion, superantigen

## Abstract

It has been posited SARS-CoV-2 contains at least one unique superantigen-like motif not found in any other SARS or endemic coronaviruses. Superantigens are potent antigens that can send the immune system into overdrive. SARS-CoV-2 causes many of the biological and clinical consequences of a superantigen, and, in the context of reinfection and waning immunity, it is important to better understand the impact of a widely circulating, airborne pathogen that may be a superantigen, superantigen-like or trigger a superantigenic host response. Urgent research is needed to better understand the long-term risks being taken by governments whose policies enable widespread transmission of a potential superantigenic pathogen, and to more clearly define the vaccination and public health policies needed to protect against the consequences of repeat exposure to the pathogen.

## 1. What Is a Superantigen?

The term superantigen was coined in 1989 [1] and defined proteins that hyper-stimulate T cells via the crosslinking of T cell receptors (TCR) with MHC Class II molecules. The definition was expanded following the discovery of B cell superantigens [2], which hyper-stimulate a large population of B cells without the crosslink. A superantigen is commonly defined as a molecule that has antigen-receptor mediated interactions with over 5% of the lymphocyte pool [3].

Put simply, superantigens are potent antigens that can send the immune system into overdrive and stimulate up to 30% of the naive T cell pool [4,5]. Reactions between superantigens and T cells may lead to a number of outcomes, including anergy, inflammation, cytotoxicity, deletion of T-cells and autoimmunity [6,7,8]. Superantigens have also been shown to impair post-vaccination memory cell responses to unrelated antigens and antagonize memory cell activation [9].

The same superantigen can produce a range of host responses. Toxic shock has been shown to develop more severely in individuals who express certain MHC Class II haplotypes which bind specific superantigens, compared with those who expressed haplotypes with lower binding affinity [10]. Responses may also be affected by environmental factors. For example, simultaneous bacterial and viral infections have been shown to increase the effects of superantigens [11]. Superantigens have been shown to impact central nervous system function and are implicated in the development of neurological conditions [12,13,14] and cardiovascular dysfunction [15,16].

Superantigens have diverse interactions with MHC class II and T–cell receptor molecules, involving a number of different interaction surfaces and stoichiometries [17,18,19]. In addition to superantigens, there are superantigen-like proteins that activate lymphocytes using mechanisms that place them outside the superantigen classification [20]. Superantigen-like proteins have been implicated in inducing thrombotic and bleeding complications through platelet activation [21,22].

SARS-CoV-2 causes many of the biological and clinical consequences of a superantigen, and we believe in the context of reinfection and waning immunity, it is important to better understand the impact of a widely circulating, airborne pathogen that may be a superantigen, superantigen-like or trigger a superantigenic host response.

## 2. Lessons from Dengue

T lymphocyte activation during dengue infection is thought to contribute to the pathogenesis of dengue hemorrhagic fever (DHF) [23]. In fact, dengue virus (DENV) causes some of the clinical characteristics seen in COVID-19, including T cell activation [23], neurological complications [24] and autoimmunity [25]. DENV-induced autoantibodies against endothelial cells, platelets and coagulator molecules lead to their abnormal activation or dysfunction [25]. A study of TCR Vβ gene usage in children with DENV infection concluded dengue is not a superantigen, but rather a conventional antigen [23]. The authors of the study cautioned their finding had limitations, but it is widely accepted DENV is a conventional antigen that causes host reactions typically associated with superantigens.

A conventional antigen can still trigger a superantigenic host response. A recent study of the response of human endogenous retroviruses (HERV) to DENV serotype 2 infection found significant differentiation in expression during infection [26]. HERVs are components of the human genome that likely originated through the historic incorporation of exogenous viruses [27]. HERVs perform important biological functions but are also implicated in the development of autoimmunity and cancer [28]. Certain viral infections have been shown to trigger HERV upregulation and autoimmunity [29]. HERVs can present proteins that act as superantigens [30]. Epstein–Barr virus (EBV) has been shown to transactivate HERV-K18, which encodes a superantigen [31]. This may have clinical implications. For example, HERV-K18 is significantly elevated in the peripheral blood of patients with juvenile rheumatoid arthritis [32].

HERV loci are upregulated by a variety of viral infections, seemingly as part of an effective innate immune response [33], but it is possible that a dysfunction in response transactivates a superantigen, which triggers an immune cascade or autoimmunity. In fact, transient elevations of HERV-K [34], and prolonged elevation of HERV-W have been found in COVID-19 patients [35,36]. HERV-W envelope protein (HERV-W-env) has been shown to induce T cell responses with superantigen characteristics [37].

## 3. Superantigens and T-Cell Dysfunction

**Figure 1:**
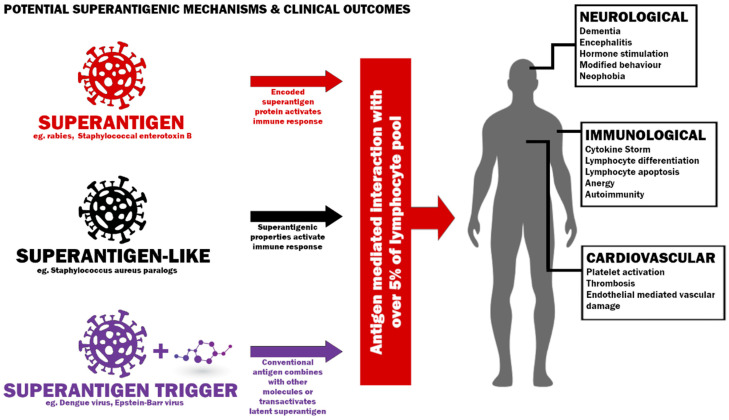
Potential mechanisms to induce a superantigenic host response and possible clinical outcomes.

Superantigens have differing effects on immature and mature CD4 and CD8 T-cells (Figure 1). Superantigens can deplete thymocytes or immature T-cells, but can hyperstimulate mature, antigen-experienced CD4s and CD8s [38]. After hyperstimulation by Staphylococcal enterotoxin B (SEB) superantigen, T-cells can enter a state of unresponsiveness known as ‘anergy’ where they fail to respond, and may sometimes subsequently enter apoptosis, or programmed cell death [39,40]. Not limited to only affecting CD4s by virtue of MHC II, superantigens can cause differentiation of naive T-cells and stimulation of CD8 memory cells from bystander activation via cytokines or from similar Vβ gene segments in their TCRs [41]. Antigen-independent activation, or bystander activation of CD8 T-cells, is a well-studied consequence of viral infection [41,42,43].

SEB superantigen activates virus-specific CD8 T-cells in vivo with both direct TCR engagement in some cases and by bystander effect [44]. This bystander stimulation is also apparent in vitro [44]. Interestingly, T-cell death elicited by superantigenic stimulation is most apparent among the T-cells activated by the bystander effect rather than activated by direct TCR engagement [45]. CD8 T-cells in which the superantigen directly stimulates per T-cell receptor β-chain retain their cytotoxic function [46]. The possibility of deletion of antiviral memory by the bystander effect warrants investigation given the involution of the thymus following puberty, as it could compromise microbe clearance [47].

Chronic exposure to superantigen could continually stimulate T-cells, keeping them in a perpetual state between anergy and hyperstimulation. Furthermore, given naive T-cells can be activated and differentiated by the bystander effect, this could manifest in an observed naive T-cell depletion in the peripheral blood where naive cells home to lymphoid tissues in individuals where new naive T-cells are not being readily generated due to thymic involution [47,48]. This effect could explain the paucity of naive T-cells in some Long COVID patients [49]. The loss of naive T-cells is a defining metric in immune aging and dysfunction. They help regulate immune responses and have the highest expansive capacity in response to antigens from cancers and infection [50,51,52].

## 4. Superantigens and Autoimmunity

Superantigens are implicated in the development of autoimmune diseases [53,54,55,56,57,58]. T-cell clones that are cross-reactive towards the endogenous host and microbial epitopes may be stimulated and migrate to tissue containing an autoantigen, a mechanism believed to play a role in the pathogenesis of rheumatic fever [59,60]. Individuals with autoimmune diseases show an increase in such T-cells in affected organs or peripheral blood [5]. Superantigens stimulate autoantibody production by bridging the MHC Class II molecule of B-cells with the TCR on T-cells [61]. Whether deletion or autoimmunity occurs seems to be a function of dose, persistence, host haplotype and severity of cytokine response [62].

Persistent subcutaneous exposure to a superantigen has been shown to cause a systemic inflammatory disease mimicking systemic lupus erythematosus (SLE) in mice [63]. Superantigens have been shown to trigger or exacerbate SLE [64]. Interestingly, HERV-E has been implicated in SLE [65,66]. HERV-E has been found to be upregulated in the bronchoalveolar lavage fluid of COVID-19 patients [67].

Insulin-dependent diabetes mellitus (IDDM) is a T-cell-mediated autoimmune disease triggered by unknown environmental factors acting on a predisposing genetic background, but there is evidence superantigen-like exposure in the form of HERV-W-env upregulation is implicated in the recruitment of macrophages in the pancreas and beta-cell dysfunction [68]. Antibodies against HERV-W-env precede or overlap with conventional IDDM antibodies in youths who are susceptible to or have the condition [69].

## 5. SARS-CoV-2 as a Superantigenic, Superantigen-like Pathogen or Superantigen Trigger

We note a recent study of SARS-CoV-2 which found immunological dysfunction following mild to moderate infection, including depletion of naive T and B-cells in individuals with Long COVID [49], and a single cell atlas which also found depletion of naive T-cells and higher levels of apoptotic T-cells in SARS-CoV-2 infection than HIV [70]. Taken together with findings on post-SARS-CoV-2 autoantibodies [71,72], presentation of MIS-C [73], activation and depletion of T-cells [74] and a rise in IDDM [75], these are suggestive of a superantigen, superantigen-like protein or triggering of a superantigenic host response as a causative agent, and further research is needed into its role and likely long-term effects, particularly since SARS-CoV-2 has been found to persist in the body months after acute infection [76,77,78,79,80,81,82]. SARS-CoV-2’s superantigenic characteristics have been implicated in MIS-C [83]. The expansion of T-cells carrying the TRBV11-2 gene, in combination with variable alpha chains, a hallmark of superantigen-mediated T-cell activation, has been reported in several studies of patients with MIS-C [84,85].

Brodin offers an energy allocation hypothesis for MIS-C, suggesting a choice in favor of disease tolerance over maximal resistance that means children are more likely to present with mild and even asymptomatic disease but might also be less efficient at viral clearance and, consequently, be more prone to some level of viral persistence and possibly other conditions linked to such viral persistence such as superantigen-mediated immune activation in MIS-C [86]. We question why such SARS-CoV-2’s superantigenic characteristics would not be assumed to apply to adults, particularly given the clinical and biological manifestations in all age groups, which reflect known prior differences between responses to superantigen exposure in adults and children. Indeed, MIS-A manifests in adults as a consequence of SARS-CoV-2 infection [87] and rare instances of Kawasaki disease are observed in adults [88,89].

The issue of whether SARS-CoV-2 contains a superantigen is not settled, but the evidence is accumulating [90,91,92,93,94,95] and SARS-CoV-2 is causing superantigen or superantigen-like clinical presentations and biomarkers. In addition to cytokine storms [96], T-cell activation and deletion [74] and presentation of MIS-C [73,97,98] (similar to Kawasaki disease, a suspected consequence of superantigen exposure [99]), those infected by SARS-CoV-2 who suffer Long COVID following infection manifest symptoms [100] typically seen in autoimmune conditions such as SLE [101,102,103], and autoantibodies [71] and antinuclear antibodies [72] have been detected in a proportion of such individuals [104].

In vitro assessments of SARS-CoV-2’s superantigen-like region may not capture the full physiological effect on the immune system in vivo. For example, lipopolysaccharide (LPS) can potentiate the SEB superantigen effect [105], which could have a synergistic effect on T cells following gut inflammation or injury via LPS translocation [106,107].

SARS-CoV-2 is known to infect gut epithelial cells [108], persist in the gut [79,109,110] and disrupt tight junctions in bronchial epithelial barriers [111]. Indeed, hospitalized non-survivors of SARS-CoV-2 infection had increased LPS detected in blood [112]. While SARS-CoV-2 may not be canonically superantigenic in vitro, the in vivo consequences may be significant due to other danger and death signals [113].

With evidence mounting that SARS-CoV-2 reactivates latent viruses such as Epstein–Barr Virus [114], cytomegalovirus [115,116] and human endogenous retrovirus [36], which are associated with superantigen expression [31,69,117,118,119], it is important to establish whether SARS-CoV-2 is a superantigen or triggering second-order superantigenic responses in susceptible individuals.

Some countries seem willing to tolerate high levels of infection provided their healthcare systems can cope. This approach is predicated on the belief a level of protective population immunity can be achieved and sustained, and the impact of reinfections will be less severe [120]. If SARS-CoV-2 contains a superantigen, superantigen-like protein or triggers a superantigenic host response, this strategy may prove a grave error. The effect of a superantigen is dependent on dose exposure, genetic predisposition, environmental conditions and immune response [6,7,12,62].

There is evidence the toxic effects of superantigens can be inhibited by specific antibodies but protection conferred seems to depend on antibody titer and exposure dose [121]. Recent evidence of a reduction in MIS-C following vaccination supports the protective role of antibodies in preventing a clinical manifestation of a superantigen or superantigen-like infection [122]; however, in the context of waning antibody titers seen following vaccination against [123] or infection [124] by SARS-CoV-2, and ongoing evolution of the virus [125], the impact of repeat exposure may be unpredictable.

Rather than proving beneficial, allowing widespread transmission of SARS-CoV-2 could be detrimental, and the growing population suffering from Long COVID [126] marked by a depletion of naive T-cells [49] may be a warning. Given the adverse impact Kawasaki disease and some autoimmune conditions can have on long-term health and longevity [127,128], national strategies that allow widespread transmission of an airborne [129] potentially superantigenic or superantigen-like pathogen that has demonstrated some evidence of persistence and can inflict repeat infections may be misguided.

## 6. Conclusions

If SARS-CoV-2 is a superantigen, superantigen-like or triggers a superantigenic host response, the unpredictable nature of a superantigen makes it particularly difficult to assess what will happen to people on repeat exposure and adds to the overall uncertainty around the long-term effects of the virus [130,131]. Urgent research is needed to confirm or refute the superantigenic nature of SARS-CoV-2, to better understand the long-term risks being taken by governments whose policies enable widespread transmission and to understand whether it is necessary to maintain consistently high levels of neutralizing antibodies to better protect against the consequences of exposure to the pathogen. It is of vital importance to definitively establish whether SARS-CoV-2 is a superantigen, superantigen-like or triggers a superantigenic host response in order to better understand the short and long-term consequences of infection. It should be noted that one of the superantigen-like motifs posited in SARS-CoV-2 is unique, and not found in any other SARS or endemic coronaviruses [83] and that according to longitudinal analysis of SARS-CoV-2, this motif appears highly conserved [132].
